# Sequential bilateral Behçet’s neuroretinitis associated with prepapillary vitreous exudate: case report

**DOI:** 10.1186/s12348-020-00226-y

**Published:** 2020-12-07

**Authors:** Imen Ksiaa, Safa Ben Aoun, Sourour Zina, Dhouha Nefzi, Sana Khochtali, Moncef Khairallah

**Affiliations:** Department of Ophthalmology, Fattouma Bourguiba University Hospital, Faculty of Medicine, University of Monastir, 5000 Monastir, Tunisia

**Keywords:** Behçet uveitis, Prepapillary exudate, Swept source OCT, OCT angiography, Case report

## Abstract

**Objective:**

To describe a case of Behçet disease (BD) uveitis manifesting with sequential bilateral neuroretinitis associated with prepapillary inflammatory vitreous exudate (PIVE).

**Material and methods:**

A single case report documented with multimodal imaging.

**Results:**

A 37-year-old man developed neuroretinitis with associated PIVE in the left eye. He was diagnosed with ocular toxoplasmosis and treated accordingly based on positive serologic testing and negative work-up for other entities, including BD. The disease course was favorable, but 1 year later a similar neuroretinitis developed in the right eye. Extraocular features of BD became evident only at the time of the second eye involvement, and the patient received corticosteroid and immunosuppressive therapy.

Swept source (SS) OCT showed at the acute phase in both eyes a typical “mushroom-shaped” prepapillary hyperreflectivity of the PIVE. SS OCT angiography (OCTA) demonstrated a corresponding prepapillary hypointense area due to shadowing effect, decreasing in size while scanning deeper layers. It also detected peripapillary retinal hypervascularity in both eyes and a sectoral area of flow signal loss in the first involved left eye. Visual acuity improved following the resolution of the PIVE and associated acute inflammatory changes in both eyes. The left eye showed residual optic disc pallor and retinal nerve fiber layer defects.

**Conclusion:**

Sequential bilateral neuroretinitis associated with PIVE may occur before other clinical features of BD become evident. SS OCT and OCTA can provide useful information for the diagnosis and management of this rare, but typical, ocular manifestation of BD uveitis.

## Introduction

Behçet disease (BD) is a multisystemic inflammatory disorder of unknown etiology characterized by recurrent inflammatory attacks [1]. Most common manifestations of BD include oral ulcers, genital ulcers, and ocular involvement. Ocular BD is typically characterized by bilateral relapsing-remitting non granulomatous panuveitis with retinal vasculitis that may result in severe vision loss [1]. Several specific eye findings may help in the early diagnosis of BD uveitis and in differentiating this condition from other infectious and non-infectious uveitis entities [2, 3]. These include shifting hypopyon, diffuse vitritis, inferior vitreous precipitates, transient superficial retinal infiltrates, occlusive retinal vasculitis, and fern-like retinal capillary leakage.

Optic disc hyperemia is a constant feature of posterior segment inflammation and optic disc hyperfluorescence is an early fluorescein angiographic finding in patients with BD uveitis [3]. Other rare forms of optic nerve involvement have been described including optic neuritis, neuroretinitis, unilateral prepapillary vitreous exudate, and ischemic optic neuropathy [2].

We herein describe a case of BD uveitis manifesting with sequential bilateral neuroretinitis associated with prepapillary inflammatory vitreous exudate (PIVE) documented with multimodal imaging, including swept source optical coherence tomography (SS OCT) and OCT angiography (OCTA).

## Case report

**Fig. 1:**
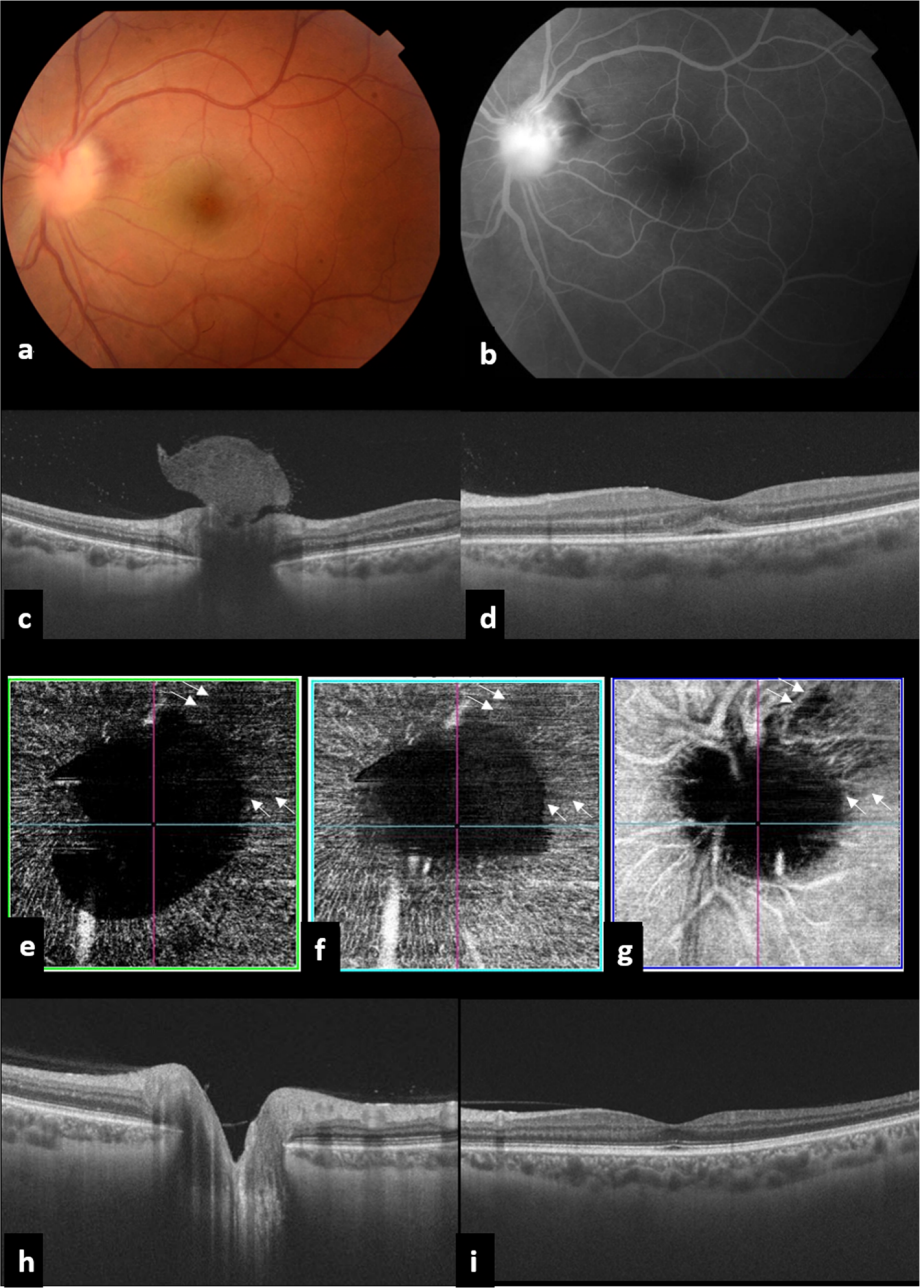
Multimodal imaging of the left eye at first presentation. **a** Fundus photograph showing a localized inflammatory vitreous exudate overlying an infiltrated optic disc and peripapillary retina with associated retinal hemorrhages **b** Late-phase fluorescein angiography demonstrating optic disc leakage and masking effect from retinal hemorrhages. **c** SS OCT of the optic nerve head showing a “mushroom-shaped” hyperreflectivity of the prepapillary vitreous exudation seen on clinical examination. **d** Macular SS OCT showing an associated shallow macular serous retinal detachment. **e**, **f**, **g** SS OCTA revealing a dark area overlying the optic disc due to blockage from the prepapillary exudate. The prepapillary hypointense area decreases in size while scanning deeper layers, reflecting the “mushroom-saphed” pattern of the prepapillary vitreous exudate. Note the presence of a superotemporal hyposignal area (arrows). One month after presentation, SS OCT shows the resolution of the prepapillary vitreous exudate and serous retinal detachment (**h**, **i**)

A 37-year-old man presented with sudden blurred vision in the left eye (LE). Best-corrected visual acuity (BCVA) was 20/20 in the right eye (RE) and 20/200 in the LE, and there was a left relative afferent pupillary defect (RAPD). Intraocular pressure was 16 mmHg bilaterally. Slit-lamp examination of the RE showed no evidence of intraocular inflammation in the anterior chamber or vitreous cavity. Results of fundus examination of the RE were unremarkable. Slit-lamp examination of the LE showed a quiet anterior chamber and 1+ vitreous cells. Fundus examination of the LE revealed a localized inflammatory vitreous exudate overlying an infiltrated optic disc and peripapillary retina with associated retinal hemorrhages (Fig. 1a). There was no evidence of retinal vascular sheathing, retinal infiltrates, or any other abnormality elsewhere in the left fundus. Late-phase fundus fluorescein angiography (FFA) of the LE showed optic disc leakage and a masking effect from retinal hemorrhages (Fig. 1b). SS OCT (DRI OCT Triton plus, Topcon, Tokyo, Japan) of the optic nerve head showed a “mushroom-shaped” hyperreflectivity of the PIVE seen clinically (Fig. 1c). There was an associated shallow macular serous retinal detachment (Fig. 1d). SS OCTA of the LE revealed a dark area overlying the optic disc and peripapillary retina due to blockage from the PIVE. This hypointense area was found to decrease in size while scanning deeper layers, reflecting the “mushroom-shaped” hyperreflectivity of the PIVE (Fig. 1e,f,g). There was a surrounding retinal hypervascularity with a sectoral superotemporal hyposignal area. Macular OCTA showed no obvious abnormal findings. Results of FFA, OCT, and OCTA of the RE were unremarkable.

The patient denied any prior history of specific systemic symptoms, and internal medicine evaluation revealed no evident features of Behçet disease. A diagnosis of presumed toxoplasmic neuroretinitis was made based on a positive anti-toxoplasmic IgG titer. Results of other investigations including indirect fluorescent antibody (IFA) test for cat scratch disease antibodies, ELISA antibodies screening for rickettsial disease, Mantoux test, chest x-ray, Treponema Pallidum Particle Agglutination TPPA assay and Venereal Disease Research Laboratory test (VDRL), angiotensin converting enzyme rate, liver enzyme tests and Pathergy test were normal or negative.

The patient was treated with the association of azithromycin (500 mg the first day, and then 250 mg/day), pyrimethamine (100 mg the first day, and then 50 mg/day), folinic acid (15 mg/day), and oral prednisone (0.75 mg/kg/day). After 4 weeks of therapy, visual acuity of the LE had improved to 20/32, and the PIVE and associated optic disc and peripapillary infiltration had completely resolved (Fig. 1h).

**Fig. 2:**
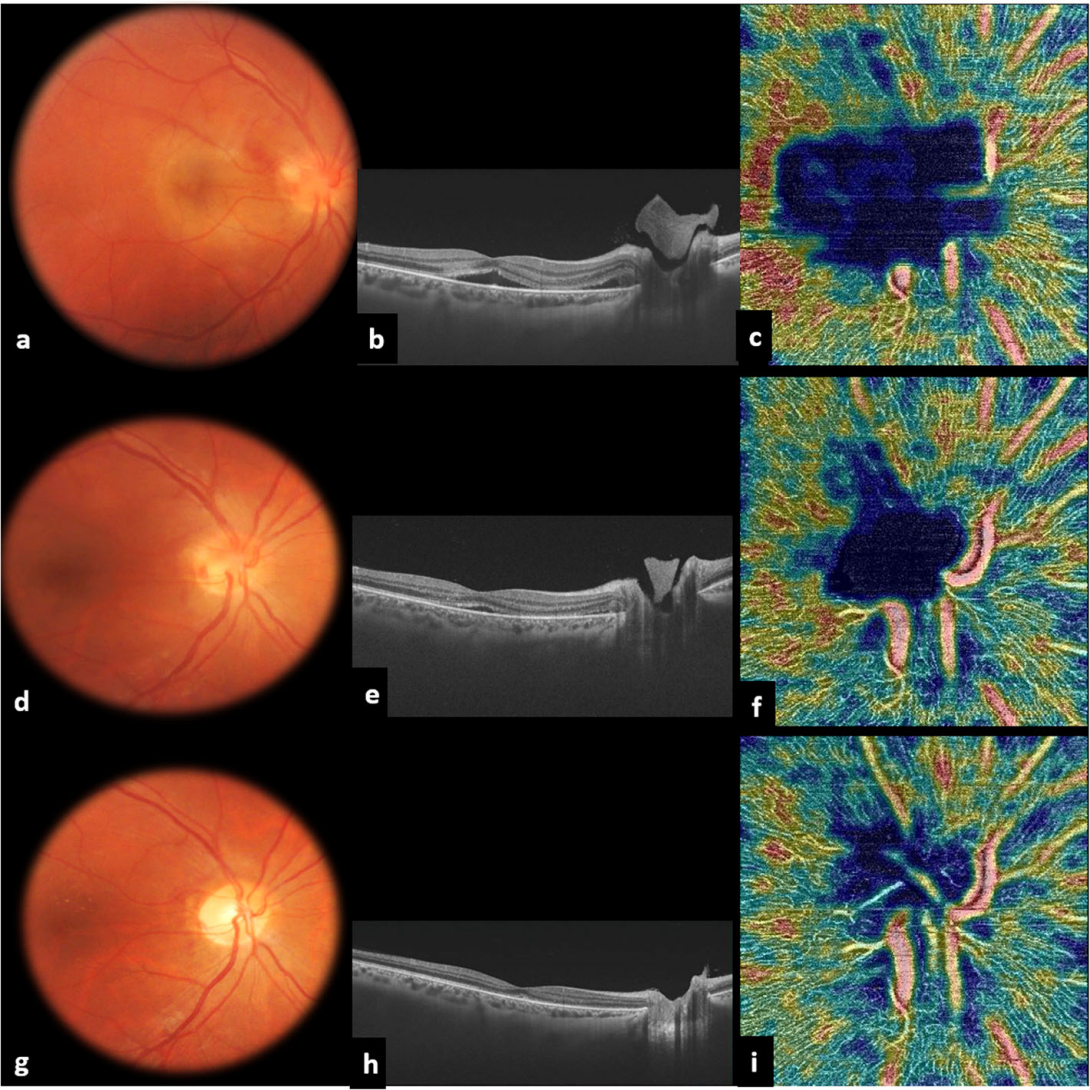
Multimodal imaging at the time of presentation for the right eye involvement. **a** Fundus photograph of the right eye showing a localized inflammatory vitreous exudate overlying an infiltrated optic disc and peripapillary retina with associated retinal hemorrhages. **b** SS OCT showing a “mushroom-shaped” hyperreflectivity of the prepapillary vitreous exudation associated with a peripapillary serous retinal detachment involving the fovea. **c** SS OCTA scan through the radial peripapillary capillary network of the right eye revealing a prepapillary hypointense area due to blockage from the prepapillary exudate. Note the presence of peripapillary retinal hypervascularity, with a peripapillary vascular density (PVD) of 11.23%. Sequential imaging follow-up at 48 h (**d**, **e**, **f**) and 1 month (**g**, **h**, **i**). Fundus photography showing the development of macular hard exudates and gradual resolution of the prepapillary inflammatory exudate and associated optic disc and peripapillary infiltration (**d** and **g**). SS OCT demonstrating gradual resolution of the prepapillary hyperreflectivity and subretinal fluid (**e** and **h**). SS OCTA showing gradual resolution of the prepapillary hypointense area and peripapillary hypervascularity, with a PVD of 9.18% at 1 month (**h**, **i**)

**Fig. 3:**
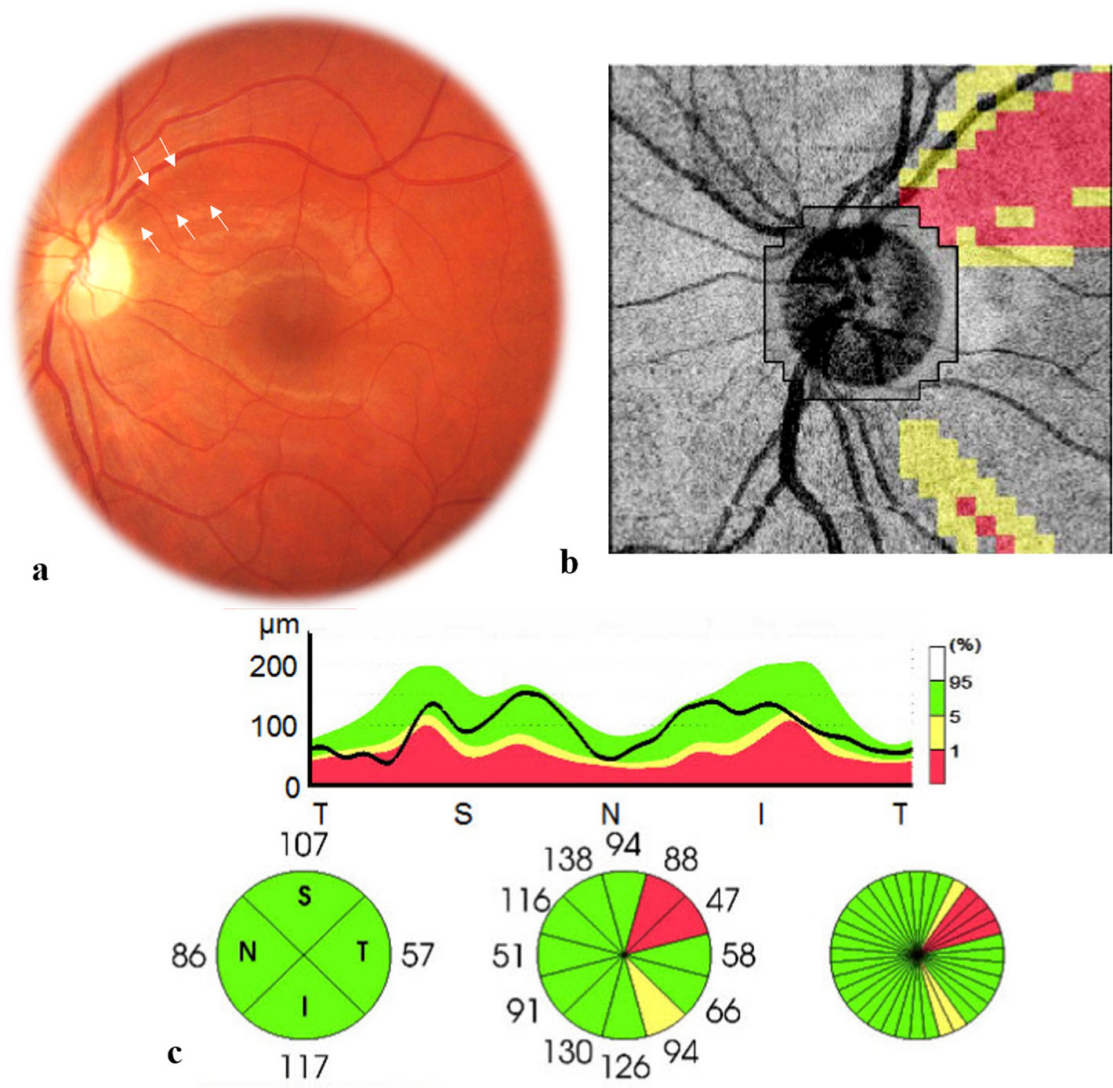
(**a**) Fundus photograph of the left eye 1 year after initial presentation showing a mild temporal optic disc pallor and superotemporal bundle retinal nerve fiber layer defects (arrows). SS OCT analysis showing corresponding superotemporal RNFL thinning and a mild subclinical inferotemporal RNLF thinning (**b** and **c**)

One year after the initial presentation, the patient complained of acute vision loss in the RE. BCVA was 20/200 in the RE and 20/20 in the LE, and there was a right RAPD. Slit-lamp examination showed a quiet anterior chamber and 2+ vitreous cells in the RE. There was no evidence of intraocular inflammation in the anterior chamber or vitreous cavity in the LE. Fundus examination of the RE revealed a localized PIVE overlying an infiltrated optic disc and peripapillary retina with associated retinal hemorrhages (Fig. 2a). There was no evidence of retinal vascular sheathing, retinal infiltrates, or any other abnormality elsewhere in the right fundus. Fundus examination of the LE showed a mild temporal optic disc pallor with superotemporal bundle retinal nerve fiber layer (RNFL) defects (Fig. 3a). SS OCT of the RE showed a “mushroom-shaped” hyperreflectivity of the PIVE seen clinically and the presence of peripapillary subretinal fluid involving the fovea (Fig. 2b). An OCTA scan in the RE through the radial peripapillary capillary network showed a hypointense area overlying the optic disc and peripapillary retina due to blockage from the PIVE. There was a surrounding peripapillary retinal hypervascularity with a peripapillary vascular density (PVD) of 11.23% (Fig. 2c). OCT retinal thickness analysis of the LE showed RNFL thinning at the superotemporal region corresponding to the RNFL defects seen clinically, with an area of subclinical inferotemporal RNLF thinning (Fig. 3b and c). Results of OCT and OCTA of the LE were unremarkable.

Physical examination by an internist revealed a recent history of recurrent oral ulcers and showed the presence of genital aphthous ulcers, pseudofolliculitis, and papulopustular lesions. A final diagnosis of BD was made based on a score of 5 according to the International Criteria for diagnosis of Behçet’s Disease [4]. The patient was treated with oral prednisone (1 mg/kg/day, followed by gradual tapering) and azathioprine (3 mg/kg/day).

Sequential follow-up examinations showed gradual resolution of acute inflammatory changes and improvement of visual acuity in the RE **(**Table 1**)**. At the one- month follow-up, BCVA was 20/20 in the RE and remained unchanged at 20/32 in the LE. There were no cells in the anterior chamber or vitreous cavity in either eye. Fundus examination of the RE showed complete resolution of the PIVE and underlying optic disc and peripapillary infiltration and the development of macular hard exudates (Fig. 2d and g). SS OCTA showed the resolution of the prepapillary hyposignal area and decrease in peripapillary hypervascularity, with a PVD of 9.18% (Fig. 2f and i). Over a further 6-month follow-up period, BCVA remained stable, the macular hard exudates in the RE gradually resolved, and no inflammatory flare-ups occurred.

**Table 1:** Succession of clinical events in the reported case

	Clinical manifestations
**Initial presentation (February, 2018)**	Neuroretinitis with prepapillary vitreous exudation, LE Initial diagnosis: ocular toxoplasmosis Antitoxoplasmic therapy
**Four weeks later (March, 2018)**	Complete resolution of the neuroretinitis and prepapillary vitreous exudation
**One year after initial presentation (February, 2019)**	Neuroretinitis with prepapillary vitreous exudation, RE Superotemporal RNLF defect, LE Oral and genital ulcers, pseudofolliculitis Final diagnosis: Behçet disease Corticosteroid and immunosuppressive therapy
**Four weeks later (March, 2019)**	Complete resolution of the neuroretinitis and prepapillary vitreous exudation

*LE* left eye; *RE* right eye; *RNFL* retinal nerve fiber layer

## Discussion

In this report, we describe a case of sequential bilateral neuroretinitis associated with prepapillary inflammatory vitreous exudate (PIVE) secondary to BD documented with SS OCT and OCTA.

Similar optic nerve involvement has been previously described as an uncommon finding in active BD uveitis [5–7]. It has been variably termed as prepapillary inflammatory vitreous opacity, exudation, condensation, or infiltrate. There are only a few reports that specifically addressed this finding in literature [5, 7]. Commonly, PIVE occurred among patients with a known history of BD [5–7]. Conversely, in our patient, the initial unilateral neuroretinitis and associated PIVE occurred in the absence of relevant extraocular signs for BD, along with a positive toxoplasmosis serology, leading to a misdiagnosis of presumed toxoplasmic neuroretinitis. One year later, a similar neuroretinitis with transient PIVE developed in the fellow eye and typical mucocutaneous lesions became evident only at that time. The patient therefore was diagnosed with definite BD and accordingly treated.

The PIVE occurred in our patient in association with marked optic disc and peripapillary infiltration. These localized optic disc changes were observed in the absence of significant inflammatory changes elsewhere in the fundus. Conversely, in previously reported cases [5–7], patients had bilateral panuveitis with retinal vasculitis concomitant to the unilateral PIVE.

Our case, consistent with previous data, shows that the PIVE and underlying optic disc and peripapillary infiltration rapidly resolve, with improvement of visual acuity.

SS OCT was useful in confirming the diagnosis of PIVE in both sequentially involved eyes by showing a characteristic prepapillary hyperreflective “mushroom-shaped” lesion. It also allowed us to detect an associated serous retinal detachment and to exclude any obvious peripapillary or subfoveal choroidal changes. SS OCTA showed at the acute phase a hypointense prepapillary area due to shadowing from the PIVE. This hypointense area was found to characteristically decrease in size while scanning deeper, reflecting the “mushroom-shaped” hyperreflectivity seen on SS OCT. SS OCTA also allowed us to detect subclinical peripapillary vascular involvement with retinal hypervascularity and increased vessel density which gradually resolved over time. In addition to that, the initially involved left eye demonstrated a peripapillary superotemporal area of signal loss which could explain the subsequent development of RNFL defects in the same eye. Localized RNFL defects have been considered in BD uveitis as an indicator of past retinal or optic nerve involvement and as a helpful ocular diagnostic clue [8]. Our results, consistent with previous data [3, 8], show that RNFL defects can be easily detectable by fundus examination, but OCT and RNFL thickness analyzer are useful in confirming the diagnosis and detecting subclinical RNFL alterations.

The bilateral sequential PIVE in our patient likely occurred due to the spread of inflammation from primary severe optic nerve infiltration [7].

The « mushroom-shaped » or « funnel-shaped » OCT pattern of BD-associated PIVE might be due to polymorphic leukocytic infiltration into the enlarged end of Cloquet’s canal [5, 7].

This report shows that bilateral sequential PIVE, indicative of severe neuroretinitis, can occur before other ocular and systemic manifestations of BD become evident, and this may lead to mistakes in diagnosis and management. Multimodal imaging including FFA, OCT, and OCTA can provide valuable information for the definitive diagnosis and appropriate management of BD-associated PIVE and underlying neuroretinitis.

## Data Availability

The datasets used and/or analysed during the current study are available from the corresponding author on request.

## Abbreviations

BD: Behçet disease
FFA: Fundus fluorescein angiography
OCT: Optical coherence tomography
OCTA: Optical coherence tomography angiography
PIVE: Prepapillary inflammatory vitreous exudate
RNFL: Retinal nerve fiber layer
SS: Swept source
